# Multicomponent intervention for controlling hypertension in the adult rural population of Pakistan: a protocol for a hybrid type III implementation-effectiveness cluster randomised controlled trial

**DOI:** 10.1136/bmjopen-2025-100365

**Published:** 2025-06-27

**Authors:** Imran Naeem, Aysha Almas, Aziz Sheikh, Catherine Hewitt, Hajra Khwaja, Saima Afaq, Saira Bukhari, Sajid Soofi, Salim S Virani, Sawera Hanif, Shiraz Hashmi, Simon Walker, Zulfiqar Ahmed Bhutta, Kamran Siddiqi, Zainab Samad

**Affiliations:** 1 Community Health Sciences Department, Aga Khan University Medical College Pakistan, Karachi, Sindh, Pakistan; 2 Aga Khan University, The Aga Khan University, Karachi, Sindh, Pakistan; 3 Medicine, Aga Khan University, Karachi, Sindh, Pakistan; 4 University of Oxford Division of Public Health and Primary Health Care, Oxford, UK; 5 Health Sciences, The University of York, York, UK; 6 Department of Health Sciences, University of York, University of York, York, UK; 7 Institute of Public Health and Social Sciences, Khyber Medical University, Peshawar, Khyber Pakhtunkhwa, Pakistan; 8 Pediatrics and Child Health, The Aga Khan University, Karachi, Sindh, Pakistan; 9 Texas Heart Institute, Houston, Texas, USA; 10 Centre for Health Economics, The University of York, York, UK; 11 Division of Women and Child Health, Aga Khan University, Karachi, Sindh, Pakistan; 12 Global Child Health, Hospital for Sick Children Research Institute, Toronto, Ontario, Canada; 13 Institute of Health Sciences, University of York, York, UK

**Keywords:** Blood Pressure, Cardiovascular Disease, Hypertension, Randomized Controlled Trial

## Abstract

**Introduction:**

Though prior trials have shown the effectiveness of community-based hypertension detection and care delivery models, their adoption and translation to practice has been slow. In this study, we will develop and test strategies for the implementation and scale-up of a proven multicomponent hypertension intervention (MCHI) in Pakistan that comprises health education, blood pressure (BP) monitoring and referrals by lady health workers (LHWs) and hypertension management by physicians in primary care settings.

**Methods and analysis:**

In this 24-month hybrid type III implementation-effectiveness cluster-randomised controlled trial, we will recruit 3000 adult hypertensive patients from two rural districts of Pakistan. We will engage public health sector managers, physicians and LHWs and use the **C**onsolidated **F**ramework for **I**mplementation **R**esearch to identify barriers and facilitators to the implementation of an already proven-to-be-effective MCHI. Using **E**xpert **R**ecommendations for **I**mplementing **C**hange and the modified Delphi technique, a set of implementation strategies addressing barriers will be identified. The strategies will be categorised as level 1 (requiring a change in processes), level 2 (requiring a change in infrastructure) and level 3 (financial restructuring). Basic health units and 250–300 households from their catchment will be considered as clusters. Clusters will be randomised in a ratio of 1:1 to intervention and control. While MCHI will be offered in both trial arms (intervention and control), the aforementioned implementation strategies will be randomised to the intervention arm only, starting with level 1 and moving to levels 2 and 3 as needed. Baseline and 6-monthly follow-up surveys, each of 6 months duration, will be conducted to collect data from the recruited participants on sociodemographics, cardiovascular disease (CVD) risk factors, CVD-related expenses and quality of life. The *primary outcome* will be the mean difference in BP-lowering medications per participant between the intervention and control arms. The primary outcome will be analysed using a linear mixed model with fixed effects for baseline value of the outcome. Additional outcomes include *implementation outcomes*: proportion of LHWs conducting health education, BP screening and monitoring, facility referrals and proportion of physicians diagnosing and treating hypertensive patients; *effectiveness outcomes:* proportion of participants with controlled BP and improved EQ-5D-5L score.

**Ethics and dissemination:**

Ethical approval has been obtained from the Ethics Review Committee of Aga Khan University Pakistan (ERC # 2023-9084-26739). Findings will be reported to: (1) study participants; (2) funding body and institutes collaborating and supporting the study; (3) provincial and district health departments to inform policy; (4) presented at local, national and international conferences and (5) disseminated by peer-review publications

**Trial registration number:**

NCT06726057.

**STRENGTHS AND LIMITATIONS OF THIS STUDY**

- The study uses a hybrid type III design, which helps ensure that the intervention’s effectiveness is considered alongside real-world applicability and is crucial for translating research into practice.
- The gradual introduction of implementation strategies to the intervention arm allows for a staged rollout that can provide insights about strategies that are most effective at different stages, offering valuable data for scale-up in similar settings.
- Being a behavioural intervention, blinding is not possible in this study, and since public sector lady health workers and physicians may have the opportunity to interact during various official meetings, there could be a possibility of contamination.
- While clusters comprising primary care facilities with surrounding catchment population of 200–250 households form a distinct geographic area, a possible spillover effect cannot be ruled out if a study participant from one cluster crosses over to another cluster facility for treatment.
- The development of strategies and delivery of intervention could be a challenge due to the involvement of multiple stakeholders from the public health sector.

## Background

 Cardiovascular diseases (CVDs) are now the leading cause of death worldwide. There has been a 138% rise in the prevalence of CVD globally from 1990–2019, a 154% increase in disability-adjusted life years (DALYs), a generic measure reflecting health loss due to morbidity and mortality and an approximate 15 per 100 000 population death rate due to CVD. South Asia has seen a significant increase in the prevalence of hypertension.^1^,^3^

Pakistan, a lower-middle-income country of approximately 230 million individuals, has seen a rapid demographic transition with CVD now a leading cause of death. According to the latest nation-wide prevalence survey, Pakistan has 46% of adults living with hypertension,^4 5^ while people as young as 35 years are being diagnosed with hypertension.^6^ In recent years, several community-level interventions have been tested and found to be efficacious in reducing blood pressure (BP) and CVD risk. These interventions have often involved using existing public health infrastructure, training, organising existing resources and delivery by non-physician healthcare workers.^7 8^ However, real-world implementation and scale-up of these proven community-based interventions has lagged.^9 10^ In the present work, to guide the implementation and scale-up of a prior proven community-based multicomponent hypertension intervention tested in Control of Blood Pressure and Risk Attenuation – Rural Bangladesh, Pakistan, Sri Lanka (COBRA - BPS) (referred hereafter as multicomponent hypertension intervention (MCHI)),^7^ we will develop and evaluate implementation strategies in conjunction with this MCHI for reducing BP in rural communities.^7^ The overall design is tailored to fulfil the following study objectives:

- To identify implementation strategies for scaling up a proven community-based MCHI using implementation research (IR) frameworks.
- To assess the effect of adding implementation strategies to community-based MCHI in improving access to evidence-based hypertension care and lowering BP in adults with hypertension.

## Methods/design

### Patient and public involvement

Community advisory panels will be formulated comprising participants from the study sites. These panels will help the research team make informed decisions with regards to study enrolment and mobilisation at community level.

### Study design

This study follows the Medical Research Council framework for developing and evaluating complex interventions.^11^ Specifically, the focus is on the implementation phase of the framework^12^ since the intervention being tested has already been shown to be effective and cost-effective in a large, high-quality multicountry randomised controlled trial (RCT).^7^ Therefore, we have chosen a hybrid type III implementation effectiveness cluster RCT to test implementation strategies/interventions while simultaneously gathering information on implementation and effectiveness outcomes.^13^

### Study setting

The study will take place in Thatta and Matiari, two rural districts located in the province of Sindh, Pakistan. Thatta has a predominantly rural population of approximately 1 million people, with 82% residing in rural areas.^14^ This district is divided into four subdistricts, also known as talukas. Matiari is also a rural district with a population of 0.77 million people, of which 76.2% live in rural areas.^15^ It is administratively divided into three talukas.

The public healthcare system in Pakistan is three-tiered and includes primary, secondary and tertiary healthcare facilities, as well as a group of community health workers known as lady health workers (LHWs). These LHWs are associated with primary healthcare facilities including basic health units (BHUs) and rural health centres. With each BHU, usually, 3–13 (an average of 5) LHWs are affiliated, and they cover its entire catchment population, resulting in approximately 200–250 households per LHW.

### Approach to address objective 1

In order to develop implementation strategies for the scale-up of the MCHI, we will first identify potential barriers and drivers to scaling up MCHI in rural public health facilities in Sindh. Then, we will select implementation strategies to address these potential barriers and strengthen potential drivers.

Since MCHI involves primary care services, hence primary care service providers, including district health officers, district non-communicable diseases focal persons, district LHW programme coordinators, LHWs, lady health supervisors (LHSs) and physicians will be invited as participants to workshops. The selection of participants will be done via nominations from the public sector district health offices. For identifying and ranking implementation strategies, provincial health department representatives, including implementation partners and policy makers, will also be engaged. A researcher trained in implementation science will facilitate these workshops. The workshops will focus first on identifying potential barriers and drivers to scale up and then on developing the implementation strategies.

#### Use of IR frameworks to develop implementation strategies

The updated **C**onsolidated **F**ramework for IR (CFIR)^16 17^ and the **E**xpert **R**ecommendations for **I**mplementing **C**hange (ERIC)^18 19^ will underpin our proposed work. Using a set of stakeholder workshops, a better understanding of potential barriers and drivers for implementing the MCHI at scale in public health facilities will be developed. The participants will receive a list of potential barriers and drivers based on CFIR construct definitions.^18 20^ They will then be requested to expand on these, identify potential barriers not included in this list and evaluate the probability of facing each barrier/driver and their possible effects. Through the modified Delphi technique, they will converge to a final list of the most important barriers/drivers after 1 to 2 iterations. Prior to the second set of workshops, based on the findings of initial discussions, a list of potential ERIC strategies that can be evaluated to address each CFIR barrier will be generated via internal team discussions. During the workshops and subsequent meetings, participants will be required to select and rank a number of implementation strategies that would be most effective in addressing each CFIR barrier/driver based on relevance, perceived importance and feasibility. Only those strategies that receive an endorsement by 50% or more participants for at least one CFIR barrier/driver will be included in the final list. Subsequently, these strategies will be categorised into three levels based on their feasibility: level 1 strategies that are easy to implement and only require a change in processes, level 2 strategies that require changes in the infrastructure and level 3 are financial strategies. Given that level 3 strategies require a change in financial mechanisms, these would be relatively difficult to implement.

### Approach to address objective 2

#### Effectiveness of the implementation strategies in conjunction with MCHI

**Figure 1:**
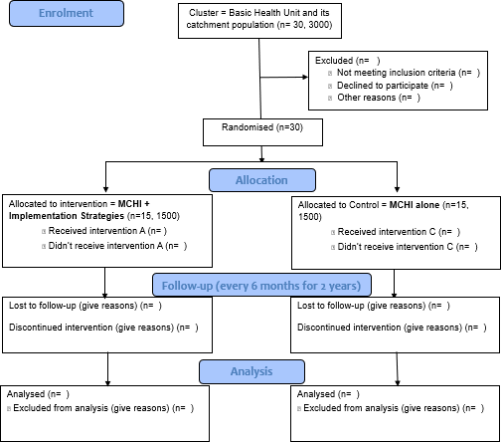
Consolidated Standards of Reporting Trials flow diagram of effectiveness-implementation hybrid trial.

To investigate the effectiveness of implementation strategies in conjunction with MCHI when delivered in the real world at scale, we will conduct a type III effectiveness-implementation hybrid trial (figure 1: Consolidated Standards of Reporting Trials (CONSORT) flow diagram of effectiveness-implementation hybrid trial).^12^ Given that MCHI targets its population through a network of LHWs connected to BHUs, a cluster-RCT design is chosen. In this trial, a cluster equates to a BHU and its catchment population. Each cluster has at least 1–2 LHWs, providing essential public health services to the catchment population on the doorstep. There are approximately 42 BHUs across both the districts. Eligible clusters will be identified using the following criteria:

- Identification of BHUs where LHWs are present and functioning.
- A list of LHWs attached to each BHU will be obtained.
- Random selection of one LHW from each cluster (BHU) and the adjacent area will be undertaken to have a catchment of 200–250 households.

About 30 clusters fulfilling the above criteria will be identified and randomised to either MCHI+implementation strategies arm (the intervention arm) or MCHI arm alone (the control arm) using a computer-generated randomisation sequence. The randomisation will be stratified by districts and assigned in a 1:1 ratio (intervention and control arms). Figure 1 shows the CONSORT flow diagram of the trial.

#### Eligibility criteria

Inclusion criteria include participants who are:

- Age ≥35 years, we have chosen a lower age cut-off compared with the COBRA trial^21^ as people at younger ages are increasingly being diagnosed as hypertensives.
- Residents of the selected clusters.
- Have hypertension defined as fulfilling any of the two following criteria:
  - Persistently elevated BP (systolic BP (SBP) ≥140 mm Hg or diastolic BP (DBP) ≥90 mm Hg) average of the last two of three readings will be considered from two separate days, where BP is measured at least 1 min apart.^22^
  - Diagnosed previously by a physician as hypertensive and/or on antihypertensive medications.

#### Exclusion criteria

Pregnant women and persons with advanced illness (eg, those receiving dialysis or with chronic liver disease leading to liver failure), cancer or any disability leading to inability to travel to the BHUs/clinics.

### Eligibility assessment and enrolment

First, we will obtain a list of all adults living in households within the selected clusters from the LHW registers. Using the inclusion criteria, all eligible adults will be identified and invited to participate, and informed consent will be obtained during a study visit (model consent form is included as online supplemental appendix I). Our trained field staff will measure BP three times using a calibrated Omron M3 HEM-7154-E automated BP monitor, with the participant in a sitting position. The readings will be taken 1 min apart. Individuals with elevated BP (SBP ≥140 mm Hg or DBP ≥90 mm Hg using average of the last two of the three readings) on the first visit will be revisited within a week for re-measurement of BP to confirm hypertension. Those with persistently high BP at the second screening visit will be invited for enrolment. Those already on antihypertensive medications will be enrolled during the first visit. All participants with elevated BP will be requested to see the physicians at BHUs; those with very high BP (SBP ≥180 mm Hg or DBP ≥120 mm Hg) will be facilitated to receive an urgent hospital appointment.

### Interventions

The MCHI will be implemented in both trial arms; however, the implementation strategies will only be introduced in the intervention arm.

#### Multi-component hypertension intervention

The MCHI is a multicomponent community-based intervention (known as COBRA) previously tested and found to be effective in a large multicountry RCT.^7^

The primary care infrastructure was utilised to deliver MCHI in the COBRA study.^7^ The components of this intervention include:

- BP monitoring and stepped-up referral to a trained physician using a checklist: at 3 month intervals, every LHW will monitor the BP of the study participants. Following this, she will be completing a BP monitoring checklist. Those with poorly controlled BP (SBP ≥160 mm Hg or DBP ≥100 mm Hg) at any visit will be referred to a trained physician for the management of hypertension. For each referral, LHW will be completing a physician referral checklist having patient details, his/her BP readings and other relevant details.
- Home health education (HHE) delivered by LHWs: the research team will train LHSs as master trainers, who in turn will conduct training of LHWs in conveying HHE sessions to study participants. Refresher training after 2 months and then annually will also be conducted to ensure rigour. The LHWs will deliver these face-to-face sessions to participants every 3 months. The HHE content is based on the manual developed as part of MCHI^7^ will have health messages focusing on non-pharmacological approaches for controlling hypertension and preventing CVD including advice on weight loss strategies, dietary modification (low salt and saturated fat intake in the diet and high consumption of fruits and vegetables), promoting physical activity and smoking cessation, seeking medical care and medication adherence. At the end of the HHE session, LHW will complete an HHE checklist with details on participants and put a checkmark against each of the areas listed above & addressed during the HHE session. All the training of LHW on HHE will be conducted by research team members experienced in community-based participatory research.
- Training of physicians in monitoring and management of hypertension and use of the checklist: the physicians will be trained in using a hypertension management manual and a treatment algorithm, both of these have been adapted from guidelines of the WHO while also taking into account the current availability of antihypertensive medication at primary care level. The physicians will receive refresher training 2 months later and annually thereafter.
- Hypertension care coordination in primary care facilities for the care of referred patient: in this real-world implementation of the MCHI, triage counters will be established in the pharmacy of the BHUs and non-physician healthcare providers such as drug dispensers will be trained in BP measurement and also provided with a digital BP monitoring device. This will facilitate the care of hypertensive individuals who present to the clinic with a physician referral checklist from the LHW. The physician in each BHU maintains a log of all hypertensive patients seen and managed.

We will use this hypertension care delivery model tested in the COBRA BPS trial and adapt it to suit the current state of healthcare at primary level as the MCHI to be implemented and scaled up in both the control and intervention arms.

#### Implementation strategies

In addition to MCHI, the intervention arm will also receive implementation strategies. While the specific implementation strategies will only be defined at the end of Phase 1, we envisage that these will be grouped as follows:

Level 1 strategies: soon after receiving training on MCHI, the staff and facilities allocated to the intervention arm will be offered the first set of strategies. While important, these would be relatively easy to implement and may require a change in processes. These may include audits and feedback, specific training and/or with increased frequency and identifying and supporting local champions, through LHWs.

Level 2 strategies: these strategies would typically require changes in the administrator infrastructure. For example, these may include making different groups of antihypertensive medications available, changing recording and reporting systems and providing clinical support tools to enhance care. Due to the nature of changes required to implement these strategies, these are more likely to be less feasible but still important.

Level 3 strategies: these would typically include financial strategies. For example, these may require financial restructuring to reward performance, financial incentives for additional services or finding new ways to finance the intervention. Such strategies tend to be most challenging to implement but likely to be effective.

Given their relative ease of implementation, we will start with level 1 strategies. Following interim analyses after the first and second follow-ups, respectively, the investigators will decide to either upgrade to the next level strategies or to continue at the current level using a set of predefined UPGRADE criteria, as shown in table 1: Criteria for upgrading the implementation strategies.

**Table 1:** Criteria for upgrading the implementation strategies

Criteria	Indicator	Source of data
Adoption	At least 80% of all participants in the intervention arm received a home visit by LHW for HHE and BP monitoring	LHW checklist validated by the participant follow-up questionnaire
Implementation	At least 80% of all participants received advice from the physicians at basic health units allocated to the intervention arm after being identified as having uncontrolled BP (SBP ≥140 mm Hg and/or DBP ≥90 mm Hg) by LHW at the first home visit	LHW and physicians’ checklists validated by participant follow-up questionnaire

BP, blood pressure; DBP, diastolic blood pressure; HHE, home health education; LHW, lady health worker; SBP, systolic BP.

If all of the above criteria are met, the current level of implementation strategies will continue. However, even if one of the criteria is not met, the implementation strategies will be upgraded to the next level.

### Control arm

The control arm will only receive the components listed as part of MCHI.

### Sample size

Each cluster is served by 1 BHU and 5 LHW on average. Each LHW serves a minimum of 100 households or 500 people (an average of 5 per household). Therefore, each cluster is estimated to have at least 2500 people. Of these, it is estimated, based on population age structure, approximately 625 would be above the age of 35 years and, of these, approximately 136 may have hypertension (25% prevalence of hypertension).^23^ If 20% refuse to participate, approximately 110 people will be eligible and ready for participation in each cluster.

We propose to assess, between the two arms, a mean difference of 0.15 in the number of BP-lowering medications/participant. If the implementation strategies are effective, over and above MCHI, those with a higher score at baseline are expected to show a bigger difference than those with a low score. Assuming a mean difference of 0.15, an SD of 0.83 (from the pooled data for Bangladesh, Sri Lanka and Pakistan by Jafar *et al*), 90% power, 5% alpha, correlation=0.5, ICC=0.02^7^ and an average cluster size of 100, then we would need to randomise 30 clusters (3000 participants).

### Outcomes and data collection

#### Primary outcome

The number of BP-lowering medications per participant at 24 months.

##### Implementation outcomes

In addition, based on the RE-AIM Framework,^24 25^ we will gather data on implementation outcomes for both the intervention and control arms (table 2: Implementation outcomes based on the RE-AIM framework).

**Table 2:** Implementation outcomes based on the RE-AIM framework

RE-AIM domain	Outcome measure	Data source
Adoption	1a. The proportion of LHWs from 30 study clusters conducting HHE sessions, monitoring BP and doing referrals of hypertensive patients to health facilities during the first 12 months2a. The proportion of physicians from 30 study clusters screening and providing hypertension management to hypertensive patients at BHU/RHC referred by LHW during the first 12 months	LHWs’ HHE and referral checklists Follow-up surveys Physician checklists Follow-up surveys
Implementation	1b. The mean number of the planned home visits/participant over 24 months (a maximum of eight visits, one every 3 months, are planned per participant) by LHW for HHE and BP monitoring	LHWs’ HHE and referral checklists Follow-up surveys
	2b. The mean number of healthcare contacts with physicians at the BHU per participant over 24 months among those identified as having uncontrolled BP (SBP ≥140 mm Hg and/or DBP ≥90 mm Hg) by LHW at one or more than one occasion during the trial	Physician checklists Follow-up surveys
Maintenance	3a. The proportion of participants receiving visits by LHWs for HHE and BP monitoring at 6, 12 and 24 months.	LHWs’ HHE and referral checklists Follow-up surveys
	3b. The proportion of participants that received advice and/or treatment from the physicians at the BHU after being identified as having uncontrolled BP (SBP ≥140 mm Hg and/or DBP ≥90 mm Hg) by LHW at 6, 12 and 24 months.	Physician checklists Follow-up surveys

BHU, basic health unit; DBP, diastolic blood pressure; HHE, home health education; LHW, lady health worker; RHU, rural health centre; SBP, systolic blood pressure.

##### Effectiveness outcomes

- The proportion of participants with BP control (SBP <140 mm Hg and DBP <90 mm Hg).
- Health-related quality of life: EQ-5D-5L range, 0–100, with higher scores indicating better health.

### Data collection: baseline and follow-up surveys

Data from the recruited study participants will be collected at baseline and then every 6 months via follow-up surveys over a period of 2 years. The duration of each survey will be 6 months. The data collection instrument, that is, the questionnaire will be transformed into an Android mobile application in local language and data will be collected digitally. All the field staff will be provided training for data collection and the use of the Android version of the questionnaire. Data will be collected on the variables including sociodemographics, medical and family history, dietary patterns, tobacco consumption (smoking and smokeless tobacco), secondhand smoke exposure, alcohol consumption, physical activity levels, sleep, quality of life (using EQ-5D-5L) and health expenditure on CVD-related medical conditions. In addition to BP measurements, height and weight will also be measured. Biological measurements will include fasting blood glucose, total serum cholesterol, low density lipoprotein and high density lipoprotein, serum creatinine and, in a subset, 24-hour urinary sodium excretion. Biological measurements will be done at baseline.

### Cost-effectiveness analysis

The cost-effectiveness of the implementation strategies will be assessed in terms of: (1) their impact on improving its adoption, implementation and maintenance and (2) their impact on patient health outcomes. Costs will be assessed from a healthcare perspective, reflecting costs of the implementation strategies, the intervention, wider healthcare resource use related to CVD and out-of-pocket payments. Cost-effectiveness will be expressed as an incremental cost per unit of effect. For their impact on health, outcomes will include life years, quality-adjusted life years and DALYs. Cost-effectiveness will be expressed as an incremental cost per unit of health outcome and incremental net health and monetary benefits based on accepted cost-effectiveness thresholds.

### Statistical analysis

Analyses will be undertaken in Stata V.17 or later versions. Significance tests will be two-sided at the 5% significance levels under intention-to-treat principles unless otherwise stated. Reporting will be in accordance with the CONSORT guidelines for cluster RCTs.

The data will be summarised descriptively by treatment group. The primary analysis will compare the number of medications per participant at 24 months between the two groups. The primary outcome will be analysed using a linear mixed model with fixed effects for baseline value of the outcome, district, distance of the cluster from the clinic, age, sex, time and interaction of time with a randomised group and random effects for the clusters and participant to account for the repeated measures by participants over time. The treatment effect at all time points will be extracted in the form of an adjusted mean difference, 95% CI and p value (with the primary being at 24 months). Continuous secondary outcomes will be similarly analysed and other outcomes by appropriate regression techniques for the type of data.

#### Trial Intervention (implementation strategies) measures to ensure fidelity

While the implementation strategies are yet to be defined, it will be important to ensure intervention fidelity by collecting data on indicators related to specific intervention strategies. The strategies could include actual versus expected number of trainings conducted, expected versus actual number of audit and feedback meetings conducted and actual versus expected number of physicians and LHWs trained over the 2-year study period.

### Data management

The principal investigator (PI) will oversee the overall management of the trial. The study management group will include the PI, co-investigators, statisticians, health economists, a qualitative expert, data managers, project managers and research assistants. The trial steering committee will consist of an independent chair (a senior professor specialising in CVD) and independent experts in implementation science and mixed methods research. The data will be collected digitally using tablets; tablets will sync with the server on internet access. Access to tablets and servers will be password-protected and will be provided to research and field team members only. Data will be encrypted for secure transfer to the server where it will be stored. Only the research team members or the authorised personnel will have access to data.

### Ethics and dissemination

Ethical approval has been obtained from the Aga Khan University (ERC # 2023-9084-26739). Findings will be reported to: (1) participating families; (2) funding bodies and institutes supporting the study; (3) provincial and district health departments to inform policy; (4) presented at local, national and international conferences and (5) disseminated by peer-review publications.

## Discussion

Hypertension is a major risk factor for CVD including stroke and ischaemic heart disease resulting in significant morbidity and mortality, globally.^26^ South Asian populations, including people from Pakistan, are at an increased risk of hypertension and CVD in both urban and rural settings,^27^ where adults of age as early as 30 years are being diagnosed with hypertension.^28^ Unfortunately, in the presence of other pressing health issues competing for human resources and the attention of service providers such as immunisation, maternal and child health and family planning, hypertension has not garnered enough attention in primary care settings in Pakistan. This leaves hypertension often not being screened and hence left untreated. Many intervention studies, particularly RCTs, despite being effective, fail to translate into practice, leading to a lack of effective uptake of findings or maintenance of intervention delivery by the provincial and national health programmes. IR aims to address this issue by producing evidence that supports the adoption and incorporation of evidence-based interventions into health policies and practices.^10 12 29^

This implementation effectiveness trial aims to engage healthcare providers at the primary care level from the designing phase to the delivery of intervention. Though the effectiveness of MCHI intervention has already been proven,^7^ we will use IR to engage stakeholders and test real-world implementation of the MCHI intervention in combination with a set of implementation strategies. We will use the CFIR framework to engage stakeholders in identifying the barriers that could potentially hinder the implementation of the intervention. Next, the use of the ERIC framework and the modified Delphi technique will have a layered strategy where health system stakeholders, including those involved in implementation at the grassroot level as well as those at the policy level, will be engaged in multiple rounds to identify implementation strategies. In developing countries, this could have its own challenges. For example, service providers at the grassroot may suggest increasing resources for successful implementation of intervention as a strategy, while policymakers at a higher level may have a broader understanding of the competing resource needs, hence terming such a strategy not implementable. Therefore, the use of the modified Delphi technique and frameworks such as the ERIC will help with identifying implementation strategies while addressing such differences in opinions before the strategies are implemented.

Finally, to evaluate the effectiveness of MCHI in combination with implementation strategies, we will use the RE-AIM framework.^25^ For policymakers and researchers, having quantifiable evidence to show the effectiveness of intervention is key for advocating and enabling its successful integration into national health programmes. RE-AIM is an extensively used IR framework to evaluate various aspects of an IR intervention including its implementation and effectiveness.

Given the challenges faced by the health system of Pakistan, this approach of integrating IR into real-world implementation of community-based interventions has the potential to strengthen hypertension care in rural primary care settings. Our trial will help identify the implementation strategies effective for real-world implementation of the MCHI intervention and which could potentially be scaled up with inputs from stakeholders including implementation partners and policy makers.

## Supplementary material

10.1136/bmjopen-2025-100365 online supplemental file 1
