# Supplementary material for: Multicomponent intervention for controlling hypertension in the adult rural population of Pakistan: a protocol for a hybrid type III implementation-effectiveness cluster randomised controlled trial
Source: BMJ Open. 2025 Jun 27;15(6):e100365. doi: 10.1136/bmjopen-2025-100365 (PMC12207168; doi:10.1136/bmjopen-2025-100365)
Supplement: online supplemental file 1 [file bmjopen-15-6-s001.doc]

**Sample Informed Consent**

This is a generic sample form to help you address most situations. Please adapt as appropriate for your research protocol and institution. *Pending rulemaking for classified human subject* *research will require additional elements of consent.*

|  |  | **Project Information** | | |  |
| --- | --- | --- | --- | --- | --- |
|  |  |  |  |  |  |
|  | Project Title: |  |  | Version & Date: |  |
|  |  |  |  |  |  |
|  |  |  |  |  |  |
|  | ERC Project No: |  |  | Sponsor: |  |
|  |  |  |  |  |  |
|  |  |  |  |  |  |
|  | Principal Investigator: |  |  | Organization: |  |
|  |  |  |  |  |  |
|  |  |  |  |  |  |
|  | Location: |  |  | Phone: |  |
|  |  |  |  |  |  |
|  |  |  |  |  |  |
|  | Other Investigators: |  |  | Organization: |  |
|  |  |  |  |  |  |
|  |  |  |  |  |  |
|  | Location |  |  | Phone: |  |
|  |  |  |  |  |  |
|  |  |  |  |  |  |

Consent document must be clearly written and understandable to subjects. The language must be non-technical (comparable to the language in a newspapers or general circulation magazine), and scientific, technical or medical terms must be plainly defined.

Informed Consent, whether oral or written, may not include language that appears to waive subjects’ legal rights or appears to release the investigators or anyone else from liability for negligence.

It must begin with the introduction of the person seeking consent. For example: “I am Dr [SAK] from Department of _ _ _, Aga Khan University and doing a research on _ _ _.” It must also include some background information on the topic of study. For example: “Disease X (Malaria) is a common disease in Pakistan, Asia and Africa, caused by a germ (parasite) spread by mosquito. It causes high grade fever. Some patients may have complications and even die. The commonly used drugs are losing their effectiveness and germs are getting resistant to it. A new drug known as [A] is supposed to be effective in treatment of disease (malaria) but there is not enough evidence that it is as good as other drugs used for treatment of disease (malaria).”

It should then state the following:

1. **PURPOSE OF THIS RESEARCH STUDY**

oInclude 3-5 sentences written in nontechnical language. “You are being asked to participate in a research study designed to...”

1. **PROCEDURES**

oDescribe procedures: “You will be asked to do...”

oIdentify any procedures that are experimental/investigational/non-therapeutic.

1. Define expected duration of subject's participation.
   1. Indicate type and frequency of monitoring during and after the study.
2. **POSSIBLE RISKS OR DISCOMFORT**

Note that these include not only physical injury, but also possible psychological, social or economic harm, discomfort, or inconvenience.

- 1. Describe known or possible risks. If unknown, state so.

1. Indicate if there are special risks to women of child bearing age; if relevant, state that study may involve risks that are currently unforeseeable, e.g., to developing fetus
2. If subject's participation will continue over time, state: “any new information developed during the study that may affect your willingness to continue participation will be communicated to you.”
   1. If applicable, state that a particular treatment or procedure may involve risks that are currently unforeseeable (to the subject, embryo or fetus, for example.)
3. **POSSIBLE BENEFITS**
   1. Describe any benefits to the subject that may be reasonably expected. If the research is not of direct benefit to the participant, explain possible benefits to others.
4. **FINANCIAL CONSIDERATIONS**
   1. Explain any financial compensation involved or state: “There is no financial compensation for your participation in this research.”
5. Describe any additional costs to the subject that might result from participation in this study.
   1. Please indicate any financial benefits to the subjects including therapeutic or diagnostic costs being covered by the study.
6. **AVAILABLE TREATMENT ALTERNATIVES**
   1. If the procedure involves an experimental treatment, indicate whether other non-experimental (conventional) treatments are available and compare the relative risks (if known) of each.
7. **AVAILABLE MEDICAL TREATMENT FOR ADVERSE EXPERIENCES**
   1. “This study involves (minimal risk) (greater than minimal risk).” In the event

that greater than minimal risk is involved, provide the subject with the following information.

- 1. If you are injured as a direct result of taking part in this research study, emergency medical care will be provided by [name] medical staff or by transporting you to your personal doctor or medical center. Indicate who will pay for this treatment.

1. **CONFIDENTIALITY**
   1. Describe the extent to which confidentiality of records identifying the subject will be maintained.

“Your identity in this study will be treated as confidential. The results of the study, including laboratory or any other data, may be published for scientific purposes but will not give your name or include any identifiable references to you.”

“However, any records or data obtained as a result of your participation in this study may be inspected by the sponsor or by AKU ERC members”.

In addition, list steps to protect confidentiality such as codes for identifying data.

1. **RIGHT TO REFUSE OR WITHDRAW**

You are free to choose whether or not to participate in this study. There will be no penalty or loss of benefits to which you are otherwise entitled if you choose not to participate. You will be provided with any significant new findings developed during the course of this study that may relate to or influence your willingness to continue participation. In the event you decide to discontinue your participation in the study, oThese are the potential consequences that may result: (list)

oPlease notify (name, telephone no., etc.) of your decision or follow this procedure (describe), so that your participation can be orderly terminated.

In addition, your participation in the study may be terminated by the investigator without your consent under the following circumstances. (Describe) It may be necessary for the sponsor of the study to terminate the study without prior notice to, or consent of, the participants in the event that (Describe circumstances, such as loss of funding.)

1. **AVAILABLE SOURCES OF INFORMATION**

oAny further questions you have about this study will be answered by the Principal Investigator:

Name:

Phone Number:

oAny questions you may have about your rights as a research subject will be answered by:

Name:

Phone Number:

oIn case of a research-related emergency, call: Day Emergency Number:

Night Emergency Number:

1. **AUTHORIZATION**

I have read and understand this consent form, and I volunteer to participate in this research study. I understand that I will receive a copy of this form. I voluntarily choose to participate, but I understand that my consent does not take away any legal rights in the case of negligence or other legal fault of anyone who is involved in this study.

Name of participant (Printed or Typed):

Date:

Signature of participant:

Date:

Signature of Principal Investigator:

Date:

Name and Signature of person obtaining consent:

Date:

***For Participants unable to read***

**Witness**:

I have witnessed the accurate reading of the consent form to the potential participants, and the individual has had the opportunity to ask questions. I confirm that the individual has given consent freely.

Witness Name: _____________________ Participant’s Thumb Print: __________________

Signature:

Date:
