# Supplementary material for: Multicomponent intervention for controlling hypertension in the adult rural population of Pakistan: a protocol for a hybrid type III implementation-effectiveness cluster randomised controlled trial
Source: BMJ Open. 2025 Jun 27;15(6):e100365. doi: 10.1136/bmjopen-2025-100365 (PMC12207168; doi:10.1136/bmjopen-2025-100365)
Supplement: Supplementary file 2 [file bmjopen-2025-100365.reviewer_comments.pdf]

PEER REVIEW HISTORY

BMJ Open publishes all reviews undertaken for accepted manuscripts. Reviewers are asked to complete a checklist review form and are provided with free text boxes to elaborate on their assessment. These free text comments are reproduced below.

ARTICLE DETAILS

Title (Provisional)

A multi-component intervention for controlling hypertension in the adult rural population of Pakistan: a protocol for a hybrid type III implementation-effectiveness cluster randomised controlled trial

Authors

Naeem, Imran; Almas, Aysha; Sheikh, Aziz; Hewitt, Catherine; Khwaja, Hajra; Afaq, Saima; Bukhari, Saira; Soofi, Sajid; S.Virani, Salim; Hanif, Sawera; Hashmi, Shiraz; Walker, Simon; Bhutta, Zulfiqar Ahmed; Siddiqi, Kamran; Samad, Zainab

---

VERSION 1 - REVIEW

Reviewer 1

Name Azmat, Syed Khurram

Affiliation Jinnah Sindh Medical University, AAPNA - Institute of Public Health

Date 02-Mar-2025

COI None

---

1) The title must mention "...adult rural population of two districts in Sindh province".

2) Why are these two districts, Thatta and Mitiari of Sindh, selected? What is the scientific reasoning behind this? There are more rural and underserved districts in Sindh province. What is the UHC status of these districts? Also, did the authors consider running this study in other rural districts in Punjab or KP provinces?

3) The protocol needs a dedicated and detailed section on "strengths and limitations", as the current provided is insufficient. Later, the author can extract 2-3 key S&L and offer it under the abstract section as part of the journal requirement.

4) The implications or future use of the findings are missing; it would be great if the authors could provide details on how they plan to use the results and use them for policy & practice.

Reviewer 2

Name Anchala, R

**Affiliation**	**Indian Institute of Public Health, Hyderabad, India , Epidemiology**
**Date**	**05-Mar-2025**
**COI**	**None**

Minor comments

1."For identifying and ranking implementation strategies, provincial health department representatives will also be engaged" – all stakeholders including implementing agencies and key policy decision makers may be included.

2. “A researcher trained in implementation science with a focus on identifying potential barriers and drivers to scale up first and then the implementation strategies.” - Some one trained in community based participatory research (CBPR) would add value to the study

3. Definition of HTN : should ideally include elevated SBP and/or DBP

4. Level 1 , 2 and 3 are well thought out and described

5. Reference for 25% prevalence in sample size calculations needs to be mentioned

6. Since level 1, 2 and 3 are being developed, Cost utility analysis may be attempted in addition to cost effectiveness analysis

7. Indicators for measuring fidelity should include a logic framework model for monitoring and evaluation of the study

8. The protocol should ideally describe individual components and methods for RE-AIM

VERSION 1 - AUTHOR RESPONSE

Reviewer: 1

Dr. Syed Khurram Azmat, Jinnah Sindh Medical University, Marie Stopes Society Pakistan

Comments to the Author:

1) The title must mention "...adult rural population of two districts in Sindh province".
Response: We have followed the PICOS criteria i.e., Population, Intervention, Comparison, Outcome & Study design to ensure a meaningful and comprehensive title. Focus of the study is on adult rural population which is also reflected in the title. Making further additions could lengthen the title, hence authors prefer to stick to the current title.

2) Why are these two districts, Thatta and Mitiari of Sindh, selected? What is the scientific reasoning behind this? There are more rural and underserved districts in Sindh province. What is the UHC status of these districts? Also, did the authors consider running this study in other rural districts in Punjab or KP provinces?

Response: The purpose of this study is to leverage the existing primary care level health infrastructure to test MCHI and implementation strategies and see which strategies work and how effective these would be. To serve this purpose, district with functional primary care infrastructure and established lady health workers program is a requirement. Additionally, research team has garnered research support from the district health administration of both the districts and is willing to cooperate for conducting the research study. Hence these two districts are chosen. Based on the results of the study, lessons learned could be scaled up in other similar rural districts of Sindh and other provinces of the country.

3) The protocol needs a dedicated and detailed section on "strengths and limitations", as the current provided is insufficient. Later, the author can extract 2-3 key S&L and offer it under the abstract section as part of the journal requirement.

Response: for S & L section, we have followed the journal requirement/guidelines for drafting protocol manuscripts. Strengths and limitations have been revised following editor's recommendations and are now more focused on study methods, page 3, lines 71-86 as follows:

Strengths and limitation

- The study uses a hybrid type III design, which helps ensure that the intervention's effectiveness is considered alongside real-world applicability, which is crucial for translating research into practice.
- The gradual introduction of implementation strategies to the intervention arm allows for a staged rollout which can provide insights into which strategies are most effective at different stages, offering valuable data for scale-up in similar settings.
- Being a behavioural intervention, blinding is not possible in this study; & since public sector lady health workers and physicians may have the opportunity to interact during various official meetings, there could be a possibility of contamination
- While clusters comprising primary care facilities with surrounding catchment population of 200-250 households forms a distinct geographic area, possible spillover effect can not be ruled out if study participant from one cluster cross over another clusters' facility for treatment
- The development of strategies and delivery of intervention could be a challenge due to the involvement of multiple stakeholders from the public health sector

4) The implications or future use of the findings are missing; it would be great if the authors could provide details on how they plan to use the results and use them for policy & practice.

Response: The suggestion has been incorporated towards end of discussion, page 13, lines 433-436 as follows:

Given the challenges faced by the health system of Pakistan, this approach of integrating IR into real-world implementation of community-based interventions has the potential to strengthen hypertension care in rural primary care settings. Our trial will help identify the implementation strategies effective for real-world implementation of the MCHI intervention and which could potentially be scaled up with inputs from stakeholders including implementation partners and policy makers.

Reviewer: 2

Dr. R Anchala, Indian Institute of Public Health, Hyderabad, India

Comments to the Author:

Minor comments

1."For identifying and ranking implementation strategies, provincial health department representatives will also be engaged" – all stakeholders including implementing agencies and key policy decision makers may be included.

Response: This has been incorporated on page 5, lines 150-151 as follows:

...implementation strategies, provincial health department representatives including implementation partners and policy makers will also be engaged.

2. “A researcher trained in implementation science with a focus on identifying potential barriers and drivers to scale up first and then the implementation strategies.” - Some one trained in community based participatory research (CBPR) would add value to the study

Response: Team members conducting the interventions trainings are trained and experienced in CBPR. Mentioned on page 8 lines 252-254 as follows:

All the trainings will be conducted by research team members experienced in community based participatory research.

3. Definition of HTN : should ideally include elevated SBP and/or DBP

Response: the definition for elevated SBP and DBP is provided in inclusion criteria on page 7, lines 201-205 as follows:

…Have hypertension defined as:

- either with persistently elevated BP (Systolic blood pressure [SBP] SBP ≥140 mm Hg or diastolic blood pressure [DBP] DBP ≥90 mm Hg) average of the last two of four readings from two separate days, where BP is measured at least one mn apart (22)

4. Level 1, 2 and 3 are well thought out and described

Response: Thank you for the feedback

5. Reference for 25% prevalence in sample size calculations needs to be mentioned

Response: A reference has been added page 10, line 307

6. Since level 1, 2 and 3 are being developed, Cost utility analysis may be attempted in addition to cost effectiveness analysis

Response: We have used the term cost-effectiveness analysis as this term is comprehensive, but as the analysis uses QALYs, we could have referred to it as a cost-utility analysis.

7. Indicators for measuring fidelity should include a logic framework model for monitoring and evaluation of the study

8. The protocol should ideally describe individual components and methods for RE-AIM

Response to comments 7, 8: following deliberations, authors have decided that it would be best to treat process evaluation and fidelity measure as a separate standalone project, hence we have removed the paragraph about process evaluation from the current manuscript.

However, description of RE-AIM' components being used for implementation outcomes is provided in table 2.

VERSION 2 - REVIEW

**Reviewer**	**2**
**Name**	**Anchala, R**
**Affiliation**	**Indian Institute of Public Health, Hyderabad, India , Epidemiology**
**Date**	**16-May-2025**

COI

All; comments have been addressed adequately
